# 629 Capturing Personal and Social Growth Through Burn Camp Experiences: An AI-assisted Analysis of Counselor Narratives

**DOI:** 10.1093/jbcr/iraf019.258

**Published:** 2025-04-01

**Authors:** Brad Jackson, Kerry Mikolaj, Nichole Schiffer, Trudy Boulter

**Affiliations:** Children’s Hospital Colorado Burn Center; Children’s Hospital Colorado; Children’s Hospital Colorado Burn Center; Children’s Hospital Colorado

## Abstract

**Introduction:**

Burn camps foster personal growth, social development, and leadership skills in young burn survivors. The American Camping Association (ACA) documents evidence for multi-dimensional growth through camp experiences. There is a need for data-driven insights into how burn camp specifically impacts personal growth and development over time. Our previous work documented self- and parent-report measures of the positive impact of burn camp. This study seeks to analyze multi-year camp counselor reports to identify key themes related to campers’ growth.

**Methods:**

Burn camp counselors complete narrative reports each year focused on campers’ independence, social skills, and adventure/exploration during intentional camp programming. We conducted a qualitative analysis of de-identified counselor reports from 2019, 2021 & 2022. Chat GPT Artificial Intelligence (AI) utilized Latent Dirichlet Allocation (LDA) to identify key themes in the data. Thematic analysis was conducted on each year’s data individually and across all years to uncover patterns and trends in campers’ personal and social development.

**Results:**

Three consistent main themes emerged across the years:

1. Confidence & Personal Growth: Camp experiences fostered leadership skills, independence, self-confidence, and resilience through challenges and new activities.

2. Social Engagement & Relationship Building: Campers formed lasting friendships and improved their social skills by working collaboratively in groups.

3. Exposure to New Activities & Adventures: New activities played a significant role in broadening campers’ experiences, helping them step outside their comfort zones while fostering curiosity, adaptability, and a sense of adventure.

There was a shift in emphasis from individual challenges and leadership in 2019 to group dynamics and outdoor experiences in 2022. This evolution reflected the growing importance of collaborative activities and social development, particularly post-2020.

**Conclusions:**

The data demonstrate that burn camp provides significant benefits for long-term personal and social development. Confidence-building activities, social bonding/peer support, and exposure to new challenges consistently emerged as key factors in fostering campers’ growth. The evolving focus towards group dynamics and outdoor experiences in more recent years highlights the role of burn camps in developing social connections and adaptability.

**Applicability of Research to Practice:**

Burn camps are crucial in promoting independence, resilience, leadership, and social skills in young burn survivors. Camps should continue emphasizing both individual and group development, with a focus on providing diverse, challenging activities that encourage personal and interpersonal growth. Camp programs can also be tailored to address the evolving needs of campers, particularly as they re-enter social settings post-pandemic.

**Funding for the Study:**

N/A

